# Objective Biobehavioral Measures Reflect Burnout States and Temporal Changes in a Nursing Population: A Prospective Observational Assessment

**DOI:** 10.3390/nursrep16010036

**Published:** 2026-01-22

**Authors:** Katelynn A. Bourassa, Bishal Lamichhane, Nicole Bartek, Chandra Bautista, Akane Sano, Alok Madan

**Affiliations:** 1 Department of Psychiatry & Behavioral Health, Houston Methodist, Houston, TX 77030, USA; blamichhane@houstonmethodist.org (B.L.); nbartek@houstonmethodist.org (N.B.); amadan@houstonmethodist.org (A.M.); 2 Houston Methodist Academic Institute, Houston, TX 77030, USA; 3 Department of Psychiatry, Weill Cornell Medical College, New York, NY 10065, USA; 4 Department of Electrical Computer Engineering, Computer Science, and Bioscience, Rice University, Houston, TX 77005, USA; akane.sano@rice.edu

**Keywords:** burnout, heart rate, sleep, digital health, nursing mental health

## Abstract

**Background/Objectives**: Nurses are at high risk for burnout. Identification of biomarkers associated with early manifestations of distress is essential to support effective intervention efforts. **Methods**: Fifty nurses from a large hospital system participated in a 30-day study of biopsychosocial factors that may contribute to burnout. Nurses wore an Oura ring that collected behavioral data and they completed a self-report burnout questionnaire at baseline and the end of the study period. Machine learning models were developed to evaluate whether objective measures could predict burnout states and changes at the end of the study period. Analyses were exploratory and hypothesis-generating for future work. **Results**: Data for 45 participants were included in the analyses. Participants with burnout had significantly higher sleep variability. Sleep measures provided 75.75% accuracy in ability to discriminate between burnout states. Heart rate-based measures better modeled changes in symptomatic components of burnout (Emotional Exhaustion, Depersonalization) over time. Heart rate-based measures provided a R-squared value of 0.13 (*p* < 0.05) (RMSE of 7.41) in a regression model of changes in Emotional Exhaustion evaluated in a leave-one-participant-out cross-validation. **Conclusions**: Sleep measures’ association with a state of burnout may reflect the longer-term manifestations of chronic exposure to workplace stress. Short-term changes in burnout symptoms are associated with disturbances in heart rate measures. Wearable technology may support monitoring/early identification of those at risk for burnout.

## 1. Introduction

Despite a plethora of organizational and individual-level interventions targeting burnout among healthcare workers, distress remains high. Nurses are especially prone to developing burnout, given unique occupational risk factors associated with their role [1]. Given the entrenched nature of workplace stressors in healthcare systems, many intervention efforts aim to bolster individual resources and are implemented once the nurse has already been suffering from burnout. To truly promote a healthy and sustainable nursing workforce, novel methods of early detection and intervention are needed.

Early detection and intervention efforts are likely hindered by the insidious nature of burnout, lack of clinical assessment tools, and the cultural norms in nursing practice. Given that burnout develops from chronic exposure to workplace stressors [2], operationalizing the syndrome as a present or absent state removes potentially meaningful variability in the burnout experience and how burnout develops over time. Recent re-conceptualizations of burnout posit that burning out likely proceeds across stages [3]. According to a model described by Leclercq and Hansez, attempts to manage stress and emotional exhaustion through mental, emotional, and physical efforts to avoid stressful situations exacerbates distress by removing individuals from needed supports, positive work experiences, and their connection to core values about their role [3]. Many individuals do not realize they are burning out until they are experiencing a state of clinical burnout and the associated negative personal and professional impacts [3]. Furthermore, the stigma of identifying as a “burned out nurse” likely increases the time from symptom progression to identification and help-seeking. Empowering nurses to monitor, identify, and use supports targeting wellbeing in the early stages of burnout development may be foundational to addressing the burnout crisis.

Wearable technologies have grown in popularity as a means of tracking biobehavioral indicators of health status. Nearly 45% of adults in the United States use a wearable device [4] to monitor physical activity, sleep quality, and cardiovascular health (e.g., heart rate, heart rate variability [HRV]) [5]. Sleep, heart rate, and HRV are commonly used proxies of the effectiveness of the body’s ability to adaptively respond to stressors [6,7] and may play a role in the development of burnout. Given that individuals are often unaware of the progression of burnout symptoms, tools that can capture objective biomarkers of distress may support earlier identification and engagement in care.

Some research suggests that several metrics routinely collected by wearables, e.g., sleep quality and HRV, are associated with distress among nurses. For example, HRV measured pre-shift is associated with burnout and subjective stress in nurses and nursing assistants [8]. Associations among burnout, poor sleep quality, latency, daytime somnolence, and sleep problems have also been reported [9,10]. However, findings across studies are equivocal. For example, recent studies using wearable technology have reported non-significant associations among sleep characteristics (e.g., latency, efficiency) and psychiatric distress in nurses [11]. Interpretability of findings across studies is limited by over-reliance on single time point assessments. Longitudinal assessments are needed to better capture general behavioral patterns and to identify important temporal changes in the relationship between biobehavioral factors and burnout.

The current study has potential relevance for nursing research and practice. First, it expands upon the existing literature by using novel methodology and statistical modeling to examine how the dynamics of sleep and cardiovascular physiology are associated with risk for developing burnout among nurses. Rather than relying on potentially biased self-report measures of sleep, the current study uses wearable technology to collect objective, real-time data over a one-month period. Additionally, machine learning improves upon traditional statistical methods by incorporating a large volume of individual data points, as opposed to averages, to characterize meaningful individual variability towards better burnout risk modeling across participants. The methodology of the current work may deepen the field’s understanding of the complex and variable trajectories to burnout. Furthermore, the study explores how physiological changes in response to stress are associated with burnout among nurses which may highlight a mechanism for earlier identification of this syndrome and development of more tailored interventions to foster wellness.

The primary aim of the study is to examine the associations of objective, longitudinal measures of sleep and heart rate-derived measures with risk for burnout among nurses. Secondary aims were to explore whether these objective measures distinguished nurses experiencing burnout from those who are not, and to model how sleep and heart rate-derived measures are associated with changes in burnout symptoms over time.

## 2. Methods

### 2.1. Participants and Recruitment

The study took place at the main campus of a large hospital system in the Southwestern United States from spring to winter 2023. Fifty nurses, in any nursing role, were recruited for this exploratory study. Nurses were eligible for the study if they were at least 18 years of age, proficient with the English language, in a nursing role at the hospital, amenable to using a wearable device for a one-month period, and not traveling more than one time zone away for the week prior to and/or during their study participation. The study was approved by the hospital’s Institutional Review Board. Procedures were consistent with the Declaration of Helsinki. Reporting of study results follows the STROBE guidelines for cohort studies (see Supplementary Material).

### 2.2. Procedures

Participation in the study consisted of two study visits and using a wearable device, the Oura ring (Gen 3; Oura Health Ltd., Oulu, Finland), for a 30-day period. During the initial study visit, participants provided informed consent, completed a questionnaire battery, and were trained to use the Oura ring. Participants were instructed to wear the Oura ring continuously. They could remove the ring when necessary to charge the battery or to complete medical procedures (e.g., MRI). At the final study visit, participants completed a follow-up questionnaire battery and returned the Oura ring. Participants were compensated USD 100 upon completion of all study activities.

### 2.3. Measures

#### 2.3.1. Symptom and Workplace Self-Report Measures

The following measures were of interest for the current analyses and were part of a larger assessment battery administered at baseline and follow-up visits. The demographics questionnaire was administered only at baseline.

Demographic questionnaire. A demographic questionnaire was developed for the study to collect personal and professional information.

Maslach Burnout Inventory-Human Services Survey (MBI-HSS). The MBI-HSS is a 22-item questionnaire designed to assess burnout symptoms among healthcare workers specifically. A total score and subscale (i.e., Emotional Exhaustion [EE], Depersonalization [DP], Personal Accomplishment [PA]) scores are calculated to characterize burnout symptoms and severity. The MBI-HSS subscales have shown good psychometric properties with high internal consistency, test–retest reliability, convergent validity, and discriminant validity [12]. Baseline study-specific internal consistency was good for EE (α = 0.90), moderate for DP (α = 0.65), and good for PA (α = 0.85). At follow-up, internal consistency was good for EE (α = 0.92), DP (α = 0.70), and PA (α = 0.72).

Patient Health Questionnaire-8 (PHQ-8). The PHQ-8 is an abbreviated form of the original 9-item questionnaire, removing the risk assessment item, assessing severity of depressive symptoms over a two-week period. Removal of item nine does not significantly impact the psychometric properties or interpretation of the measure [13].

Generalized Anxiety Disorder-7 (GAD-7). The GAD-7 is a 7-item measure of severity of anxiety symptoms over a two-week period. The measure has excellent internal consistency and good test–retest reliability [14].

#### 2.3.2. Physiological Data

The Oura ring is a wearable device worn on any finger that collects physiological data using infrared photoplethysmography, 3-D accelerometer, and negative temperature coefficient [15]. This analysis focused on the heart rate and sleep data obtained during the monitoring period, given their relevance to characterizing burnout dynamics. We used the 5 min heart rate resolution data provided by Oura, which is mostly obtained during the nighttime, where high-quality assessments can be obtained [16]. The heart rate data used for the analysis capture resting/night-time physiology rather than work stress reactivity (94.4% of heart rate data obtained were between 8 PM and 8 AM in our deployment). Given the limited sample size, we did not explore the fine-grained HRV measures that are also available per 5 min of nighttime measurements during sleep. Sleep assessments considered in our analyses consisted of total sleep duration as well as two ratios: the REM sleep ratio (REM duration/total sleep duration) and the deep sleep ratio (deep sleep duration/total sleep duration). These were computed from the long sleep periods as classified by the Oura assessment, avoiding assessments from naps (sleep duration between 15 min and 3 h, with some detected sleep phases) and short rest (no sleep phases detected in short sleep periods). Sleep phase labeling is performed by Oura automatically (proprietary algorithm) to provide the per-sleep phase duration for each sleep assessment. While the gold standard for sleep phase assessment is polysomnography (PSG), the Oura ring has shown high accuracy in sleep phase assessment in comparison to PSG [15]. Oura-based assessments possibly provide important insights into the sleep composition at a coarse-grained level such as total sleep phase durations in a sleep period, if not at a fine-grained level such as the epoch-by-epoch sleep phase assessments, compared to the gold standard of PSG.

### 2.4. Data Analysis

#### 2.4.1. Participant Groups

Participants were categorized into two groups: (1) those whose MBI-HSS scores were at or above the burnout threshold at both the baseline and follow-up (the ABT group; persistently high burnout), and (2) those whose MBI-HSS scores were below the burnout threshold at both the baseline and follow-up (the BBT group; persistently low burnout). The cut points for EE (≥27) and DP (≥13), based on normative data in a United States healthcare worker population [17], and recommendations by Dyrbye and colleagues for categorizing burnout [18] were used to categorize burnout in this study. Crossing threshold on either or both of the EE or DP scales was considered a burnout state. Prior research demonstrated that EE and DP scores distinguished patients with burnout from those without in an outpatient setting [19]. For analyses associating burnout state with objective measures, only participants with at least a week of monitoring data were included to ensure that sufficient longitudinal observations were available.

Participants who moved across the burnout thresholds at baseline and follow-up (the transition group) were not considered for group analysis of biobehavioral data associated with burnout state. It was not possible to localize when the transition in symptoms occurred, and localization is required to correctly associate the observed biobehavioral data (sleep and heart rate) to a burnout state. For example, participants with differing states in the baseline and follow-up, their data obtained during the monitoring period would be a mix of manifestations of both burnout/non-burnout states. Thus, a burnout state label cannot be assigned for this group. However, this group can still contribute to modeling the changes in burnout scale scores.

#### 2.4.2. Objective Measures from Physiological Data

The heart rate measurements were obtained on a fine-grained scale (per 5 min window) from the Oura ring. Sleep data, on the other hand, were obtained per day (as earlier described; the sleep measures analyzed were total sleep duration and REM/deep sleep ratio). To have the heart rate measurements on the same temporal scale as the sleep assessments, we obtained the mean, standard deviation, and entropy of the heart rate measurements per day, characterizing each day of assessment. The entropy was computed with the Shannon entropy definition as implemented in the Scipy library (v 1.15.3) of Python (v3.13.5). These measures characterize the average value, variability, and distribution to summarize the overall state and transitions of naturally varying heart rate during sleep.

After standardizing the scale (heart rate and sleep data on a daily temporal scale), each monitoring day is represented by six-dimensional sleep/heart rate features: total sleep duration, deep sleep ratio, REM sleep ratio, HR mean, HR std, and HR entropy. Since each participant was monitored for multiple days, with nominal monitoring period of a month, the distribution of the daily features across the monitoring period for each participant was characterized statistically with the first four moments of the data. The mean, standard deviation, skewness, and kurtosis of the daily features were computed resulting in 24-dimensional features characterizing each participant’s sleep (12 features) and heart rate data (12 features) over the monitoring period. Thus, the longitudinal measurements are summarized to participant-level measures for cross-sectional analysis across participants (matching the per-participant outcomes of burnout state at the follow-up and overall changes in burnout scale scores).

Missing or incomplete daily Oura ring recordings (e.g., device removal for charging or procedures) were handled by excluding days with less than six hours of range for valid heart rate measurements or missing sleep assessments. Only those participants with at least seven days of valid monitoring days were included in the analysis. The distribution of number of valid monitoring days across participants is provided in the Appendix A (Table A1). Higher-order moments (skewness, kurtosis) should be interpreted with caution for participants with fewer days of monitoring because skewness and kurtosis require sufficient observations for stable estimation.

#### 2.4.3. Objective Measures Associated with Burnout States

To examine associations among the objective wearable data and burnout states, *t*-test/Mann–Whitney U test (for normal/non-normal data assessed using the Shapiro–Wilk test) were used to examine differences in sleep and heart rate measures between the ABT and BBT groups. Corrections for multiple comparisons were not applied as this study is geared to generate hypotheses for future confirmatory studies with larger sample sizes. The predictive power of the objective measures to identify the ABT or BBT grouping of the participant was assessed using a machine learning model. A support vector machine (SVM) with an RBF kernel as the classification model was used (scikit-learn library v1.6.1 in Python). The SVM outperformed the random forest and logistic regression-based models that were evaluated for comparison. The SVM-based classification model was evaluated in a leave-one-participant-out (LOPO) fashion. The input features were normalized within the training set with standard scaling (removing mean and scaling to unit variance). The test set was normalized with the scaling parameters from the training set. The regularization parameter C in SVM was identified within the training set with nested cross-validation (grid search for C on a logarithmic scale from 10^−4^ to 10^4^). The gamma parameter was set to scale, where the value is set according to the number of features and the variance of the data. A full sweep-based parameter search for gamma was not performed to limit the combinatorial parameter search space when both C and gamma have to be tuned, given the limited dataset size. No class weight balancing was used as the class imbalance was not severe. The model was evaluated with classification accuracy and compared with baseline classification accuracy obtained by always predicting the dominant class. The feature importance was assessed using permutation feature importance where a feature’s contribution to classification performance is obtained based on the performance degradation of the classification model when that feature’s values are randomly shuffled.

#### 2.4.4. Objective Measures Associated with Burnout Dynamics

The EE and DP scores obtained from the MBI-HSS questionnaire captured the fine-grained changes in burnout state and the associated burnout risk of the participants. The changes in EE and DP scores were obtained as DeltaEE and DeltaDP scores, computed as EE/DP score at follow-up minus EE/DP score at intake (thus, single outcome per participant for cross-sectional analysis). Then, Pearson’s correlation coefficient between objective measures and DeltaEE and DeltaDP was computed to characterize the association of objective measures with the reported changes in burnout scores. The analysis was performed in the entire participant sample, irrespective of their burnout states or observed transitions across states.

A machine learning-based regression model in a LOPO fashion was used to assess if the objective measures are predictive of EE/DP score changes. With the objective measures and current EE/DP scores as input, a random forest (RF)-based regression model was trained with the hyperparameters tuned with nested cross-validation in the training set (grid search for number of estimators in {3,5,7,9,11,21} and minimum samples per leaf in {2,3,4,5}). The RF-based model outperformed the support vector machine-based regression and linear regression model evaluated for comparison. The regression model was evaluated with the correlation of the predicted score change and the true score change, as well as the R-squared values of the prediction.

## 3. Results

### 3.1. Participant Demographics

**Figure 1:**
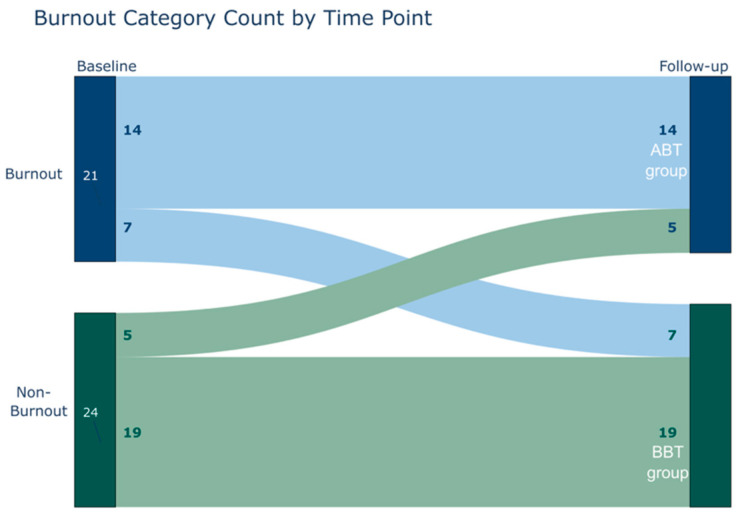
Number of participants in each burnout category at baseline and follow-up. The burnout category is based on the EE (Emotional Exhaustion) and DP (Depersonalization) scores on the MBI-HSS (Maslach Burnout Inventory—Human Services Survey) questionnaire.

Forty-five participants had at least one week of objective monitoring data and were included in the final sample. See Table 1 for demographic characteristics. Of the overall sample, 14 participants scored above the threshold for burnout at both assessments (the ABT group), 19 participants scored below the threshold for burnout at both assessments (the BBT group), and 12 moved between the categories. This is demonstrated in Figure 1. Of the 21 participants above the threshold for burnout at baseline, 17 had higher than threshold scores in EE, 3 in DP, and 4 in both the EE and DP. The 19 participants with above the threshold for burnout at follow up were composed of 14 with higher threshold in EE, 1 in DP, and 4 in both EE and DP scores.

### 3.2. Objective Measures Associated with Burnout States: Differences in ABT and BBT Groups

**Figure 2:**
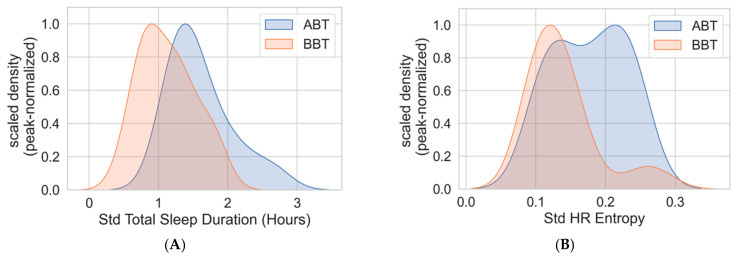
(**A**) Distribution of the sleep duration variability (characterized by the standard deviation of total sleep duration) in the ABT (above threshold for burnout at both assessments) and BBT (below threshold for burnout at both assessments) groups. The distribution is shown with scaled density plots obtained from kernel density estimation. The ABT group had higher variability of sleep duration over the monitoring duration (*p* < 0.01). (**B**) Distribution of the HR (heart rate) entropy variability in the ABT and the BBT group (difference *p* < 0.05).

The ABT group had significantly higher sleep variability (characterized by the standard deviation of total sleep duration across monitoring days) than the BBT group (1.60 +/− 0.45 h for ABT vs. 1.13 +/− 0.39 h for BBT, *t* (31) = 3.29, *p* < 0.01, Cohen’s *d* of 1.14 (95% CI [0.36, 1.91]). See Figure 2. For context, the transition group had the standard deviation of total sleep duration of 1.38 +/− 0.49 h, which falls between the average values for the ABT and the BBT group. The standard deviation of total sleep duration had a statistically significant association with the average EE scores (Pearson’s correlation coefficient of 0.39, *p* = 0.02) but not the average DP scores (Pearson’s coefficient of 0.21, *p* = 0.23). In the heart rate-derived measure, the standard deviation of the daily HR entropy was also higher for the ABT group (0.18 +/− 0.05, median 0.18) compared to the BBT group (0.14 +/− 0.05, median 0.13) *U* = 192, *p* < 0.05), Cohen’s *d* of 0.77 (95% CI [0.03, 1.51]). For the transition group, the standard deviation of the daily HR entropy value was 0.14 +/− 0.05, similar to the values for the BBT group.

Examination of the predictive power of biobehavioral measures revealed that the sleep-based measures provided substantially higher classification accuracy (i.e., ABT or BBT groups) compared to the baseline, indicating that sleep measures likely predict burnout state. See Table 2. The classification accuracy was 75.75% with a corresponding balanced accuracy of 74.24%, specificity of 64.28%, sensitivity of 84.21%, and ROC-AUC of 0.85. Five participants in the ABT group were falsely misclassified as belonging to the BBT group. Three participants in the BBT group were falsely classified as belonging to the ABT group. Permutation feature importance showed that the top three important features for classification were the standard deviation of total sleep duration, the skewness of the deep sleep ratio, and the mean total sleep duration. The classification accuracy was always substantially higher than the baseline (majority class as prediction outcome) when the thresholds for EE/DP to identify the burnout state were changed (EE threshold set to 27 and DP swept from 11 to 15; DP threshold set to 13 and EE threshold swept from 25 to 29).

### 3.3. Objective Measures Associated with Dynamic States in Burnout: Modeling Changes in EE and DP Scores

While differences in sleep and heart rate measures between the ABT and BBT groups show how persistent burnout states likely reflect in the current biobehavioral state of the participants, understanding how these measures are associated with changes in the burnout scale scores (EE and DP) can show if and how objective wearable data captures transient states in burnout scale scores for better risk modeling. The sample included participants with improving (negative DeltaEE/DeltaDP), worsening (positive DeltaEE/DeltaDP), or stable burnout scores (zero DeltaEE/DeltaDP). The ranges for DeltaEE and DeltaDP were −15 to +25 and −11 to +12, respectively. DeltaEE was moderately associated with DeltaDP, with a Pearson’s correlation coefficient of 0.51 (*p* < 0.001).

Interestingly, the DeltaEE and DeltaDP were not associated with changes in depression severity (DeltaPHQ-8, computed as PHQ-8 total score at the end of the study minus the PHQ-8 total score at the start of the study) or anxiety (DeltaGAD-7 computed as GAD-7 total score at follow-up minus GAD-7 total score at baseline). Pearson’s correlation coefficients between DeltaEE and DeltaPHQ-8/DeltaGAD-7 were 0.08 (*p* = 0.56) and −0.16 (*p* = 0.27), respectively. Similarly, Pearson’s correlation coefficients between DeltaDP and DeltaPHQ-8/DeltaGAD-7 were −0.04 (*p* = 0.77) and −0.09 (*p* = 0.52), respectively.

The association between objective sleep and heart-rate measures and DeltaEE and DeltaDP (unadjusted for potential confounders) is shown in Table 3. Only the association with the top five objective measures (based on the absolute correlation coefficient value) is shown for brevity. Several heart rate and sleep-based measures are associated with changes in EE and DP scores. Of the heart rate and sleep-based measures, the heart rate-based measures are more commonly and strongly associated with DeltaEE and DeltaDP.

**Figure 3:**
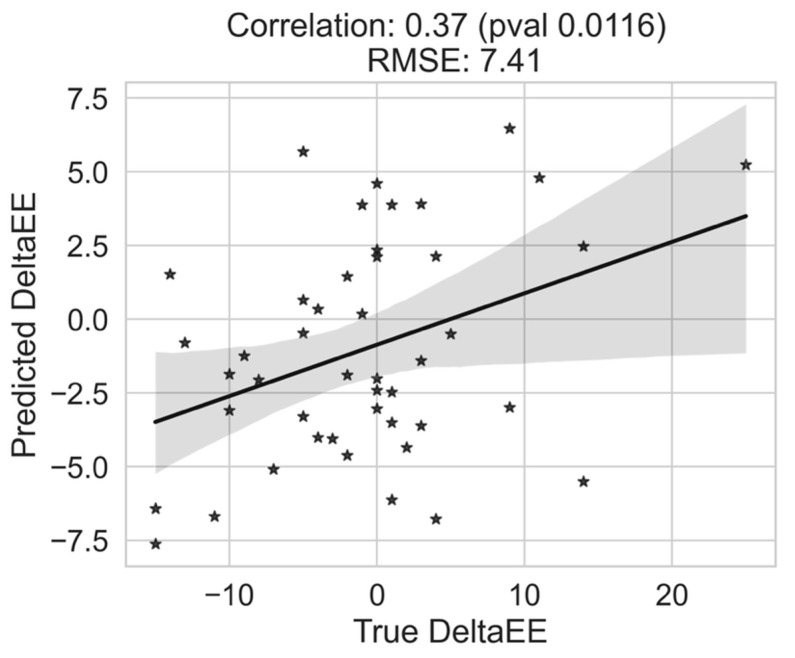
Prediction of DeltaEE (changes in emotional exhaustion—EE—score at follow-up compared to the baseline) using the heart rate measures in a leave-one-participant-out prediction using a random forest-based regression model. The grey region represents the confidence interval for the linear regression fit. The predicted changes in Emotional Exhaustion are moderately correlated with the true Emotional Exhaustion changes. Some notable participants whose DeltaEE could not be modeled well (e.g., participant with true DeltaEE of >20) is likely because this is a less representative participant in the training set.

To analyze if the observed association of the objective sleep and heart rate measures with changes in EE and DP scores has utility for burnout risk modeling, we evaluated machine learning-based regression modeling (out-of-sample regression prediction in LOPO fashion). The regression modeling of DeltaEE scores using heart rate and sleep-based measures is shown in Table 4 and the prediction for the best modeling using heart rate measures is shown in Figure 3. Unlike the DeltaEE modeling, the DeltaDP modeling did not show significance in regression modeling-based prediction with sleep, heart rate, or combined measures.

## 4. Discussion

The incidence of burnout among nurses has been deemed a public health crisis. Current assessment and intervention tools are limited and primarily based on subjective awareness of distress. Objective measures of the body’s stress response could characterize biobehavioral changes associated with distress and risk for burnout among nurses. Wearable technology provides a non-invasive and real-time platform for collecting biobehavioral data. The results of the current study suggest that sleep and heart rate measures can serve as biomarkers of burnout-related distress among nurses. This study contributes to the current literature by addressing methodological limitations (e.g., subjective assessment of sleep, single time point assessment) and offers a more nuanced picture of how biobehavioral factors are associated with burnout over time.

The findings of the current study revealed considerable variations in burnout state and associated risks at the individual level. A notable 27.6% of the nurses moved between above and below threshold of burnout over the month period, suggesting that nurses can have quick onset, remission, or relapse of burnout symptoms over a relatively short time period. This finding aligns with a previous study that reported significant variability in burnout among healthcare workers over time (with timepoint of assessment being a significant predictor in longitudinal regression models [20]). Interestingly, previous work has argued that burnout symptom domains, EE and DP, may progress and remit at different speeds. For example, emotional exhaustion is recognized as a symptom cluster that is more responsive to change than depersonalization. As emotional exhaustion is also hypothesized to be the earliest stage of burnout development [3], focusing identification and intervention efforts here may be more effective for prevention of burnout than targeting later stages. While current recommendations for burnout mitigation include assessment every few months [21,22], the results of the current study suggest that more frequent assessment may be necessary to capture evolutions in the burnout process.

Interestingly, biobehavioral markers of distress were differentially associated with burnout state and stage in the current study. Participants with at/above threshold levels of burnout had significantly higher sleep variability compared to those scoring below the threshold. Sleep measures also distinguished between burnout states at follow up. Unlike sleep, heart rate-based measures did not distinguish between participants with at/above and below threshold burnout. The heart rate-based measures did not aid sleep measures for classifying burnout states, even reducing the classification accuracy (Table 2), likely resulting from possible overfitting in the training set. Better fusion strategies for improved classification should be explored in future work with a larger sample size. Heart rate-based measures, however, were associated with changes in EE, although not DP scores over the study period. The heart rate-based measures resulted in an R^2^ of 0.13 (RMSE of 7.41) in an out-of-sample validation to predict EE score changes, indicating a moderate effect size of the association. That the heart rate-based measures could explain 13% of variability in the EE score change, though meaningful, calls for richer representations and better modeling for a more accurate prediction model. Changes in EE and DP scores were independent of changes in depressive and anxiety symptoms, supporting the assertion that burnout and mood-related psychiatric distress are distinct constructs [23] that may require unique intervention. Sleep metrics were not associated with changes in the EE or DP scores. These results, although preliminary, point to the hypothesis that short-term/acute changes in burnout states are associated with cardiovascular functioning, and longer-term states of burnout are associated with sleep disruption. This pattern is consistent with physiological frameworks in which sleep architecture reflects cumulative dysregulation over longer periods, whereas cardiac autonomic markers respond more acutely to daily psychosocial stressors. These mechanisms may explain why sleep measures were associated with persistent burnout state, while heart-rate-based indices were associated with short-term changes. Future studies should further investigate these preliminary findings obtained from our study with sample size.

The current study also supports a growing body of literature suggesting that wearable technology could be a tool to support individual wellness in the workplace. A recent randomized controlled trial demonstrated that physicians who wore smartwatches had lower burnout and higher resilience scores at follow up compared to physicians who did not wear a smartwatch [24]. Additionally, national estimates of wearable adoption shows that nearly 45% of adults use a wearable device, with higher rates of adoption among Hispanic/Latino and Black adults [4]. As historically marginalized groups are more likely to experience discrimination in the workplace that contributes to burnout [24] and less are likely to use formal mental health supports [25,26], wearable technology may provide a unique opportunity to support the wellbeing of nurses of underrepresented identities.

The findings have significant implications for healthcare organizations and nursing staff. First, the findings demonstrate the dynamicity of burnout risk among this population and highlight the value of objective biobehavioral monitoring to monitor risk. Nurses were amenable to using a wearable device and had adequate adherence, supporting the potential for non-invasive wearable devices in burnout detection and intervention. The association between heart rate-derived measures and temporal changes in EE suggest that heart rate-derived measures, obtained via wearable devices, could serve as a readily assessable indicator of increasing distress and need for intervention. If given this feedback in real time, nurses could more effectively support their wellbeing while navigating workplace challenges; for example, earlier implementation of measures to address workplace stressors (e.g., reduce length of shifts, workload changes) and personal (e.g., sleep, mindfulness, social support) could slow or reverse the burnout process. Given the non-invasive nature and relative affordability of wearables, and the organizational benefits of mitigating burnout, healthcare organizations may consider offering access to wearable technology to their nurses to promote a culture of wellness. Thus, the findings of this study suggest that monitoring physiological changes via wearable technology could be one tool to efficiently assess risk for burnout among nurses.

### 4.1. Limitations

The results of the study should be interpreted within the context of several limitations related to small sample size, the single-site study, and limited contextualization of the data. First, given the small sample of participants from one hospital system, though the modeling used LOPO cross-validation for assessing robust out-of-sample results, the results may not generalize to other populations of healthcare workers. Second, data on shift characteristics (e.g., night, rotating, length) were not collected, which is a limitation of the study given the demonstrated impact of alternative shifts on sleep. Third, given the small sample size, the effects of professional role (e.g., intensive care nurse, administrative roles), years of practice, baseline mental health, and other demographic factors (e.g., gender) were not explored. Not assessing the impact of the aforementioned variables as important contextual factors in the analysis is a limitation to be addressed in future work. As the previous literature has demonstrated associations among professional and individual characteristics and risk for developing burnout, studies with larger and more variable samples are needed to better elucidate for whom and in what context physiological risk factors for burnout are most salient. Fourth, the findings of this exploratory study should be interpreted in light of the moderate internal consistency of the DP subscale of the MBI-HSS at baseline in the current sample. Finally, dichotomizing burnout removed potentially meaningful variability in the burnout experience. The small sample size precluded the ability to use more complex statistical analyses, such as latent profile analysis, to classify stages of burnout. Larger studies with more advanced statistical modeling are needed to understand individual variability in the burnout process and related risk and protective factors.

Despite limitations, the presented work demonstrated innovative use of wearable devices, employed robust methodology with LOPO cross-validation, provided clear clinical relevance with wearable’s potential for early burnout detection, and demonstrated distinction between long-term and short-term burnout risk indicators.

Future work should further explore possible relationships among biopsychosocial factors and well-being among nurses who “recover” from burnout and those who reach clinical levels of burnout over time. Studies of this nature are needed to explore possible relationships among biopsychosocial factors and the various stages of the burnout process and recovery. Future work should also explore the impact of receiving health feedback from a wearable device on the burnout process among nurses.

### 4.2. Conclusions

Methods to identify nurses at risk for burnout earlier in the process of burning out are needed to improve prevention and intervention efforts. This study demonstrates the potential utility of monitoring physiological changes, specifically heart rate-derived measures, to detect burnout in earlier stages (e.g., emotional exhaustion). Wearable technology may be a relatively accessible way for nurses to monitor changes in their health status that may be associated with burnout-related distress. Providing nurses with the means to more effectively monitor their emotional and physical health and offering accessible wellness resources may be a burnout mitigation strategy for healthcare organizations.

## Figures and Tables

**Table 1:** Demographic characteristics of the entire sample, nurses above burnout threshold, and nurses below burnout threshold.

	Total Sample *n* (%)	ABT *n* (%)	BBT *n* (%)
Number of Participants	45	14	19
Age (years) M, SD	39.4 ±10.3	39.4 ± 9.9	41.3 ± 11.0
Gender			
*Female*	36 (80.0)	11 (78.6)	16 (84.2)
*Male*	9 (20.0)	3 (21.4)	3 (15.8)
Ethnicity			
*Non Hispanic*/*Latino*	36 (80.0)	11 (78.6)	15 (78.9)
*Hispanic*	5 (11.1)	2 (14.3)	2 (10.5)
*Latino*	3 (6.7)	0 (0)	2 (10.5)
Race			
*Asian*	14 (31.1)	4 (28.6)	6 (31.6)
*Black/African American*	10 (22.2)	4 (28.6)	5 (26.3)
*White*	17 (37.8)	4 (28.6)	7 (36.8)
*Other*	4 (8.9)	2 (14.3)	1 (5.3)
Marital Status			
*Never Married*	15 (33.3)	5 (35.7)	4 (21.1)
*Married*	25 (55.6)	7 (50.0)	12 (63.2)
*Divorced*	4 (8.9)	1 (7.1)	3 (15.8)
*Widowed*	1 (2.2)	1 (7.1)	0 (0)
Education			
*Some College*	1 (2.2)	1 (7.1)	0 (0)
*Associate’s degree*	2 (4.4)	1 (7.1)	1 (5.3)
*Bachelor’s Degree*	30 (66.7)	10 (71.4)	13 (68.4)
*Master’s Degree*	10 (22.2)	2 (14.3)	4 (21.1)
*Doctoral Degree*	1 (2.2)	0 (0)	0 (0)
Years of Practice			
<*3 years*	6 (13.3)	2 (14.3)	4 (21.1)
*3*–*6 years*	11 (24.4)	6 (42.9)	2 (10.5)
*6*–*10 years*	9 (20.0)	1 (7.1)	4 (21.1)
*10*+ *years*	19 (42.2)	5 (35.7)	9 (47.4)

Note. ABT = Above Burnout Threshold; BBT = Below Burnout Threshold; One ABT participant did not report ethnicity; One BBT participant did not report level of education; 12 participants transitioned burnout state between baseline and follow-up and their demographic characteristics are not separately shown here in the table.

**Table 2:** Classification of the ABT and BBT participants using the objective measures of sleep and heart rate.

Measures	Accuracy (%)
Baseline	57.57%
Sleep measures	75.75%
Heart rate measures	48.48%
Sleep + Heart rate measures	45.45%

Note. The baseline performance is obtained from a majority class classifier, one that always assigns participants to the BBT category (the dominant class).

**Table 3:** Top five objective measures associated with changes in the EE and DP score during the monitoring period.

	Correlation	*p*-Value
Correlation with DeltaEE		
Kurtosis—HR SD	0.53	0.0002
Skewness—HR SD	0.48	0.0008
SD—HR Entropy	0.40	0.0065
Mean—HR Entropy	−0.33	0.0283
Skew—Deep Sleep Ratio	−0.25	0.0923
Correlation with DeltaDP		
Kurtosis—HR SD	0.39	0.0073
Skewness—HR SD	0.33	0.0255
Mean—HR mean	−0.32	0.0315
Mean—HR SD	−0.29	0.0543
SD—HR Entropy	0.27	0.0709

Note. HR = heart rate; SD = standard deviation. Results are based on the *p*-value of correlation coefficient associated with changes in the EE and DP score during the monitoring period, i.e., the DeltaEE and DeltaDP scores.

**Table 4:** Prediction of DeltaEE using objective measures of sleep and heart rate.

Features	Correlation of Prediction (*p*-Value)	R^2^ Values
Sleep measures	−0.16 (*p*-value: 0.2873)	−0.29
Heart rate measures	0.37 (*p*-value: 0.0116)	0.13
Sleep + Heart rate measures	0.34 (*p*-value: 0.0226)	0.10

Note. The heart rate measures provided a significant prediction in a leave-one-participant-out (LOPO) cross-validation evaluation.

## Data Availability

The datasets presented in this article are not publicly available due to the sensitive nature of the variables collected.

## Supplementary Materials

The following supporting information can be downloaded at: https://www.mdpi.com/article/10.3390/nursrep16010036/s1, STROBE Statement—Checklist of items that should be included in reports of cohort studies.

## Public Involvement Statement

No public involvement in any aspect of this research.

## Guidelines and Standards Statement

The manuscript adheres to the STROBE guidelines for cohort studies.

## Abbreviations

The following abbreviations are used in this manuscript:

HR	Heart rate
HRV	Heart rate variability
EE	Emotional exhaustion
DP	Depersonalization
PA	Personal Accomplishment
REM sleep	Rapid eye movement sleep
SVM	Support vector machine
RF	Random forest
LOPO	Leave one participant out

## Appendix A

**Table A1:** Distribution of number of valid monitoring days for the study participants where sleep and heart rate measurements from Oura ring were available for analysis.

Number of Monitoring Days	Number of Participants
30	13
29	10
28	2
27	8
26	3
25	2
24	2
22	2
21	1
18	1
16	1
13	1
8	1
5	2
